# Supplementary material for: C. elegans TFIIH subunit GTF-2H5/TTDA is a non-essential transcription factor indispensable for DNA repair
Source: Commun Biol. 2021 Nov 25;4:1336. doi: 10.1038/s42003-021-02875-8 (PMC8617094; doi:10.1038/s42003-021-02875-8)
Supplement: Supplementary file 2 — Supplementary Information [file 42003_2021_2875_MOESM2_ESM.pdf]

Supplementary figure 1

a

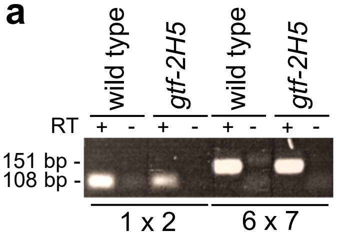

b

primer pair	wild type	*gtf-2H5*
1 x 2	++	+
1 x 3	++	-
1 x 4	-	+
1 x 5	-	-
2 x 6	-	-
6 x 7	++	++

c

```
                10          20          30          40          50          60
1    ATGGTGAACGTGAAGAAGGGAGTGTTGGTGACGAGTGATCCGGCTTTTCGTCAACTTCTG
1     M  V  N  V  K  K  G  V  L  V  T  S  D  P  A  F  R  Q  L  L

                70          80          90         100         110         120
61   ATCCATTTGGACGACTCCAGACAGCTCGGATCCAAGTTCATTGTCAGGGCACTTGTGCCA
21    I  H  L  D  D  S  R  Q  L  G  S  K  F  I  V  R  A  L  V  P

               130         140         150         160         170
121  CGTGGCGCAAGATGCAACGCATCGAGTTTCGCATTGGATTCCCGAATGTAG
41    R  G  A  R  C  N  A  S  S  F  A  L  D  S  R  M
```

d

```
                 alternative start                        E55X
          M1T                  L21P                         R56X
human     MVNVLKGVLIECDPAMKQFLLYLDESNALGKKFIIQDIDDTHVFVIAELVNVLQERVGELMDQNAFSLTQK*
C. elegans MVNVKKGVLVTSDPAFRQLLIHLDDSRQLGSKFIVRELDDTHLFIEKEIVPMLENKVEQIMENMNPEAVDK*
                                        tm6330 truncation
```

Supplementary figure 1 Mutant *gtf-2H5* cDNA and expression of GTF-2H5 and GTF-2H1
a Independent RT-PCR from Fig 1A, confirming reduced levels of cDNA in *gtf-2H5* animals and showing that in these animals the mRNA of upstream DNA repair gene *helq-1* is normally produced, ruling out that any phenotypes observed in *gft-2H5* animals are due to an effect on HELQ-1. **b** Table to indicate which cDNA PCR products were detected with primer pairs shown in Fig 1 in wild type and *gtf-2H5* animals. '++' indicates that a strong and '+' that a weak PCR band was detected. **c** Shown is the sequence of the ORF found in the cDNA of the *tm6360* allele. Black nucleotide sequences are from the first exon of *gtf-2H5* while red colored sequences are reverse sequences derived from the B0353.1 gene. The putative translation is also indicated. **d** Shown are the wild type human and *C. elegans* GTF2H5/TTDA protein sequences with all known human patient and *C. elegans* mutations indicated in different colors. M1T mutates the start codon, which is thought to lead to usage of the alternative start codon indicated in red.

Supplementary figure 2

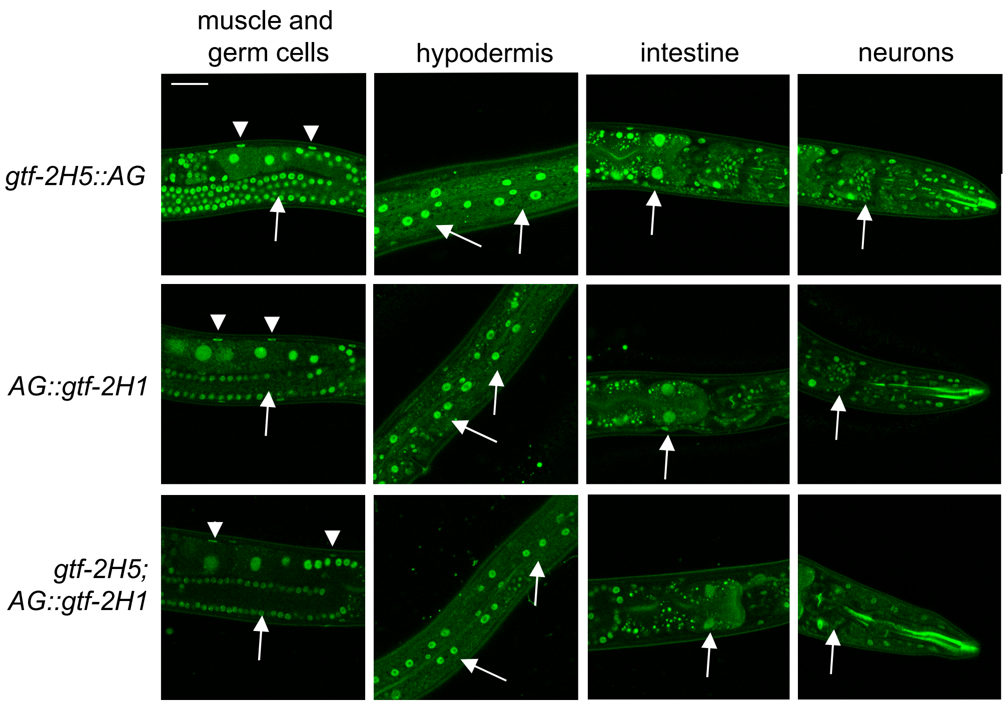

Supplementary figure 2 Expression of GTF-2H5 and GTF-2H1

Representative confocal images showing fluorescence of GTF-2H5::AG (upper panels) and AG::GTF2H1 in wild type background (middle panels) and *gtf-2H5* background (lower panels). Tissues indicated are muscle (arrow heads) and germ cells, epidermal cells, intestinal cells and head neurons (arrows). Scale bar: 25 μm.

Supplementary figure 3

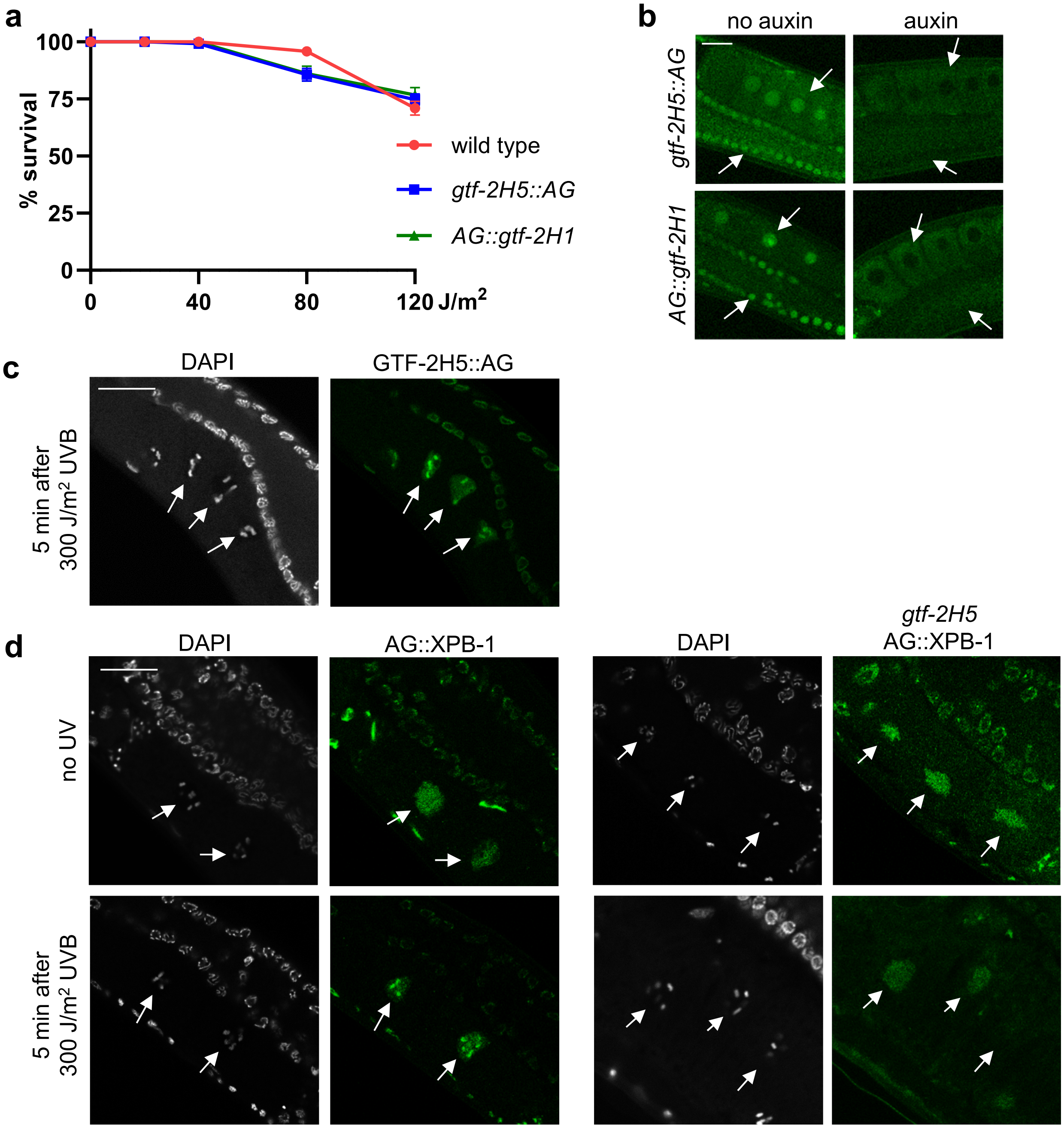

Supplementary figure 3 *gtf-2H5::AG* and *AG::gtf-2H1* knock-in and TFIIH recruitment to DNA damage **a** L1 larvae survival assay after UVB irradiation of wild type and *gtf-2H5::AG* and *AG::gft-2H1* knock-in animals. The percentages of animals that developed beyond the L2 stage (survival) are plotted against the applied UVB doses. Results are plotted as average with SEM (error bars) of ten experiments. **b** Representative confocal images showing efficient depletion of AID- and GFP-tagged GTF-2H1 and GTF-2H5 in animals expressing TIR1 under control of the *sun-1* promoter grown on 1 mM auxin for 24 h. Arrows indicate nuclei of germ cells. Scale bar: 20 µm. **c** Representative confocal images showing recruitment of GTF-2H5::AG to UVB-damaged chromosomes, visualized with DAPI (indicated in white), in oocytes of fixed wild type animals, 5 min after 300 J/m^2 UVB irradiation. Paired homologous chromosomes in oocytes are indicated with arrows. Scale bar: 20 µm. **d** Representative confocal images showing nuclear localization of AG::XPB-1 to UVB-damaged chromosomes, visualized with DAPI (indicated in white), in oocytes of fixed wild type (left) and *gtf-2hH5* (right) animals, without UV and 5 min after 300 J/m^2 UVB irradiation. Paired homologous chromosomes in oocytes are indicated with arrows. Scale bar: 20 µm.

Supplementary figure 4

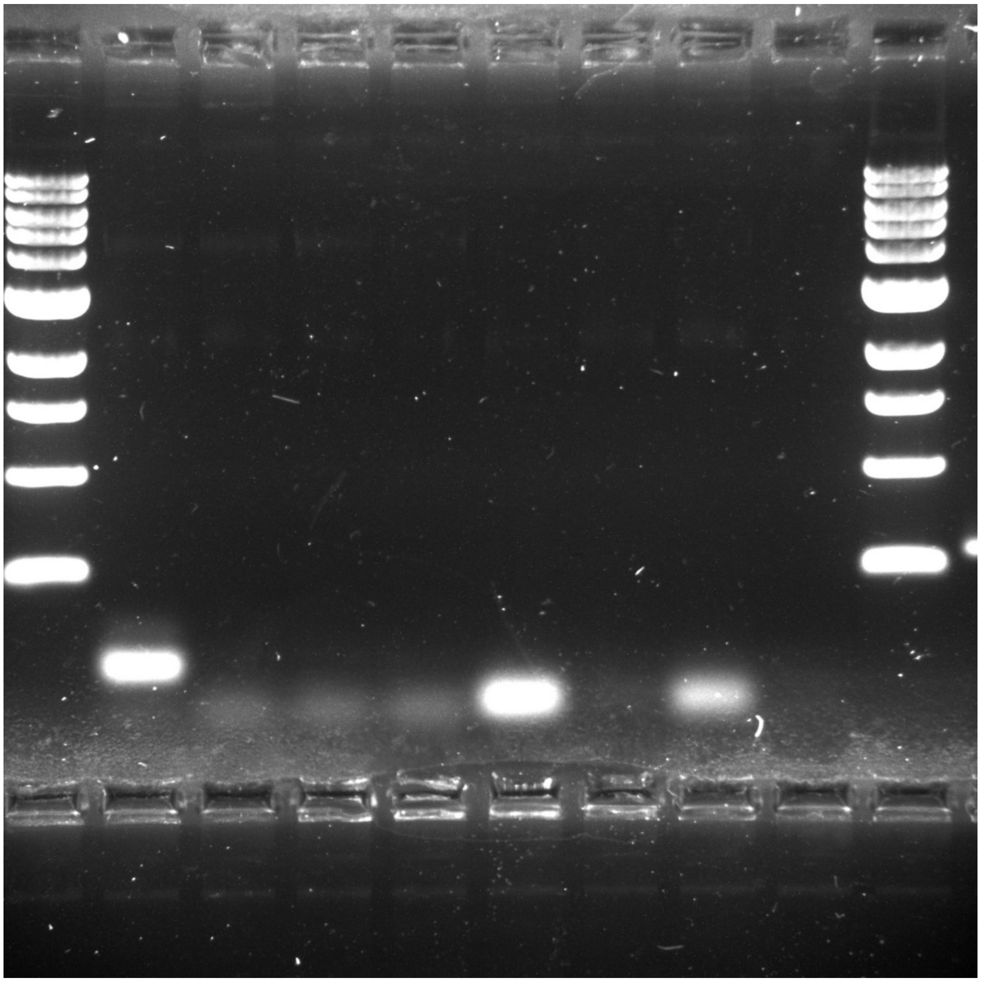

Supplementary figure 4 Agarose gel of genotyping PCR
Depicted is the full image of the agarose gel shown in Figure 1b, showing the PCR with primer pairs 1x2 or 1x3, as indicated in Figure 1a, on cDNA of wild type or *gtf-2H5* animals.

Supplementary table 1

strain	genotype
CA1199	*ieSi38[[P(sun-1)::TIR1::mRuby] IV*
CA1202	*eSi57[P(eft-3)::TIR1::mRuby] II; ieSi58 [P(eft-3)::AID::GFP] IV*
GJ1566	*xpa(ok698)I 6xoc*
HAL94	*gtf-2H5(tm6360) III*
HAL203	*gtf-2H5(tm6360) xpb-1(emc58[AID::GFP::xpb-1]) III*
HAL204	*xpb-1(emc58[AID::GFP::xpb-1]) III*
HAL237	*gtf-2H5(emc73[gtf-2H5::AID::GFP]) III; ieSi38[P(sun-1)::TIR::mRuby] IV*
HAL240	*gtf-2H5(emc73[gtf-2H5::AID::GFP]) III*
HAL242	*gtf-2H5(tm6360)III; gtf-2H1(emc202[AID::GFP::gtf-2H1]) IV*
HAL243	*ieSi38[P(sun-1)::TIR1::mRuby] gtf-2H1(emc202[AID::GFP::gtf-2H1]) IV*
HAL254	*ieSi57 [P(eft-3)::TIR1::mRuby] II; gtf-2H5(tm6360)III; ieSi58 [P(eft-3)::AID::GFP] IV*
HAL504	*gtf-2H1(emc202[AID::GFP::gtf-2H1]) IV*
N2	wildtype

Supplementary table 2

	gene	sequence	sequence	primer Fig 1
**RT-PCR**	*gtf-2H5*	GGTGAACGTGAAGAAGGGAG		1
	gtf-2H5	CCTGACAATGAACTTGGATC		2
	gtf-2H5	GTCAACAGCCTCCGGGATTC		3
	B0353.1	GTTGCATCTTGCGCCACGTG		4
	B0353.1	GAGCTCGCCTACTATCAATTG		5
	helq-1	CAACGACTTTCTGAAGCTGCC		6
	helq-1	CCCAAACGTCGAGTTCTTCC		7
**RT-qPCR**	*cdc-42*	TCCACAGACCGACGTGTTTC	AGGCACCCATTTTTCTCGGA	
	pmp-3	GTTCCCGTGTTCATCACTCAT	ACACCGTCGAGAAGCTGTAGA	
	gtf-2E1	GGTGGATGAGATTCCGGAGG	GCTCGCATTATGTGGTAGACG	
	gtf-2H5	GGTGAACGTGAAGAAGGGAG	CCTGACAATGAACTTGGATC	
