# Supplementary material for: C. elegans TFIIH subunit GTF-2H5/TTDA is a non-essential transcription factor indispensable for DNA repair
Source: Commun Biol. 2021 Nov 25;4:1336. doi: 10.1038/s42003-021-02875-8 (PMC8617094; doi:10.1038/s42003-021-02875-8)
Supplement: Supplementary file 3 — Description of Additional Supplementary Files [file 42003_2021_2875_MOESM3_ESM.pdf]

Description of Additional Supplementary Files

File name: Supplementary Data 1
Description: Annotated table of proteins identified by mass spectrometry analysis of AG::GTF-2H1 immunoprecipitation in wild type and gtf-2H5 animals.

File name: Supplementary Data 2
Description: Source data for blot (Fig 1b) and graphs in Figs 1-6.
